# Supplementary material for: Practice of Awake Prone Positioning in Critically Ill COVID-19 Patients—Insights from the PRoAcT–COVID Study
Source: J Clin Med. 2022 Nov 26;11(23):6988. doi: 10.3390/jcm11236988 (PMC9739110; doi:10.3390/jcm11236988)

Supplement to:

# Practice of Awake Prone Positioning in Critically Ill COVID–19 Patients––Insights from the PRoAcT–COVID Study

**Willemke Stilma [1,2,*,†], Christel M. A. Valk [1,†], David M. P. van Meenen [1,3], Luis Morales [4,5], Daantje Remmelzwaal [1], Sheila N. Myatra [6], Antonio Artigas [7,8], Ary Serpa Neto [9,10], Frederique Paulus [1,2] and Marcus J. Schultz [1,11,12,‡,§] on behalf of the PRoAcT–COVID Collaborative Group**

[1] Department of Intensive Care, Amsterdam University Medical Centers, Location ‘AMC’, 1105 AZ Amsterdam, The Netherlands
[2] Center of Expertise Urban Vitality, Faculty of Health, Amsterdam University of Applied Sciences, 1105 BD Amsterdam, The Netherlands
[3] Department of Anesthesiology, Amsterdam University Medical Centers, Location ‘AMC’, 1105 AZ Amsterdam, The Netherlands
[4] Servei de Medicina Intensiva, Hospital Universitari Sant Pau, 08025 Barcelona, Spain
[5] Translational Research Laboratory, Institut d’Investigació i Innovació Parc Taulí I3PT, Universitat Autònoma de Barcelona Sabadell, 08208 Barcelona, Spain
[6] Department of Anaesthesiology, Critical Care and Pain, Tata Memorial Hospital, Homi Bhabha National Institute, Mumbai 400012, India
[7] Intensive Care Department, CIBER Enfermedades Respiratorias, Parc Tauli University Hospital, 08208 Sabadell, Spain
[8] Autonomous University of Barcelona, 08193 Sabadell, Spain
[9] Australian and New Zealand Intensive Care, Research Center (ANZIC-RC), Monash University, Melbourne 3800, Australia
[10] Department of Critical Care Medicine, Hospital Israelita Albert Einstein, São Paulo 05652-900, Brazil
[11] Centre for Tropical Medicine and Global Health, Nuffield Department of Medicine, University of Oxford, Oxford OX3 7LG, UK
[12] Mahidol–Oxford Tropical Medicine Research Unit (MORU), Faculty of Tropical Medicine, Mahidol University, Bangkok 10400, Thailand
* Correspondence: w.stilma@amsterdamumc.nl
† These authors contributed equally to this work.
‡ PRoAcT–COVID, Practice of Adjunctive Therapies in COVID-19 study.
§ Collaborative authors are presented in Appendix A.

Word counts: 311 words (abstract) and 3018 words (body of the text)
Inserts: 2 figures and 4 tables
Appendices: 1 online appendix, with 1 table and 2 figures

**Content:**

Table S1 Baseline characteristics of the matched cohort p. 3

Figure S1 A-D. Unmatched and matched analysis Kaplan Meier curve for ICU and hospital LOS p. 4

Figure S2 A-D. Unmatched and matched analysis Kaplan Meier curve for ICU and hospital mortality p. 5

**Table S1. Baseline characteristics of the matched cohort**

| | Overall<br>N = 245 | Prone Positioning<br>N = 70 | Standard Care<br>N = 175 | *P* |
|---|---|---|---|---|
| Age, years (median [IQR]) | 66 [59 – 73] | 66 [60 – 74] | 66 [59 – 73] | 0.726 |
| Male gender, N (%) | 172 (70.2) | 45 (64.3) | 127 (72.6) | 0.260 |
| BMI, kg/m$^2$ (median [IQR]) | 28.4 [25.4 – 32.3] | 29.3 [26.0 – 32.2] | 27.8 [25.3 –32.4] | 0.348 |
| SAPS II score (median [IQR]) | 44 [33 – 54] | 46 [36 – 54] | 44 [32 – 54] | 0.558 |
| Do-not-intubate order, N (%) | 6 (2.4) | 0 (0.0) | 6 (3.4) | 0.267 |
| Comorbidities | | | | 0.463 |
| Arterial hypertension, N (%) | 97 (39.6) | 30 (42.9) | 67 (38.3) | 0.606 |
| Cardiovascular disease, N (%) | 60 (24.5) | 10 (14.3) | 50 (28.6) | 0.029 |
| Heart failure, N (%) | 11 (4.5) | 2 (2.9) | 9 (5.1) | 0.661 |
| COPD or astma, N (%) | 43 (17.6) | 12 (17.1) | 31 (17.7) | 1.000 |
| Diabetes mellitus, N (%) | 70 (28.6) | 16 (22.9) | 54 (30.9) | 0.273 |
| Chronic kidney disease, N (%) | 21 (8.6) | 5 (7.1) | 16 (9.1) | 0.801 |
| Malignancy, N (%) | 14 (5.7) | 3 (4.3) | 11 (6.3) | 0.761 |
| Neuromuscular disease, N (%) | 7 (2.9) | 2 (2.9) | 5 (2.9) | 1.000 |
| Obstructive sleep apnea, N (%) | 16 (6.5) | 4 (5.7) | 12 (6.9) | 0.967 |

Abbreviations: BMI, Body Mass Index; COPD, Chronic obstructive pulmonary disease; SAPS, Simplified Acute Physiology Score.

**Figure S1 A-D. Unmatched and matched analysis Kaplan Meier curve for ICU and hospital LOS**

**A: ICU discharge unmatched, B: Hospital discharge unmatched, C: ICU discharge matched, D: Hospital discharge matched**

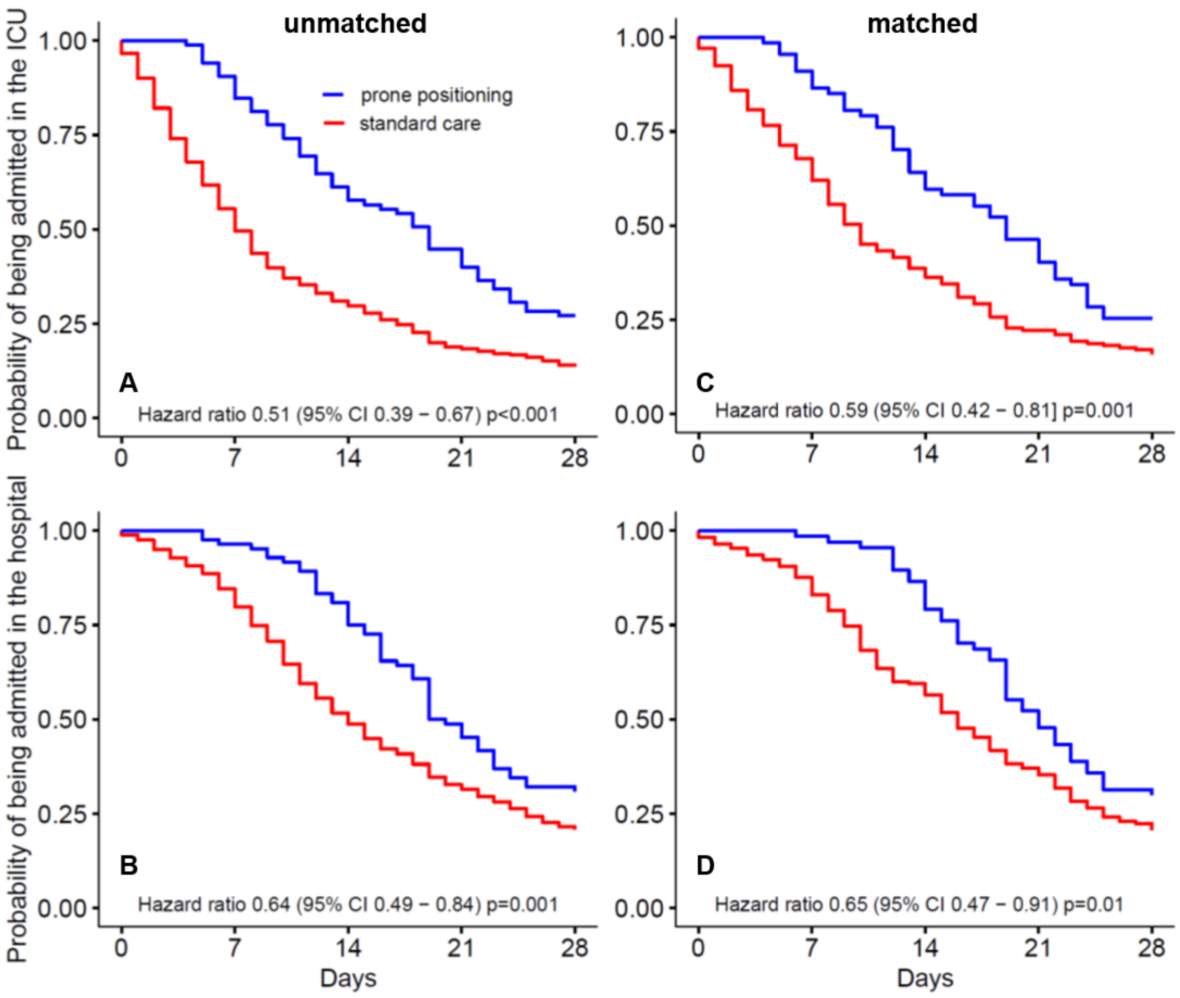

**Figure S2 A-D. Unmatched and matched analysis Kaplan Meier curve for ICU and hospital mortality**

**A: ICU mortality unmatched, B: Hospital mortality unmatched, C: ICU mortality Matched, D: Hospital mortality Matched**

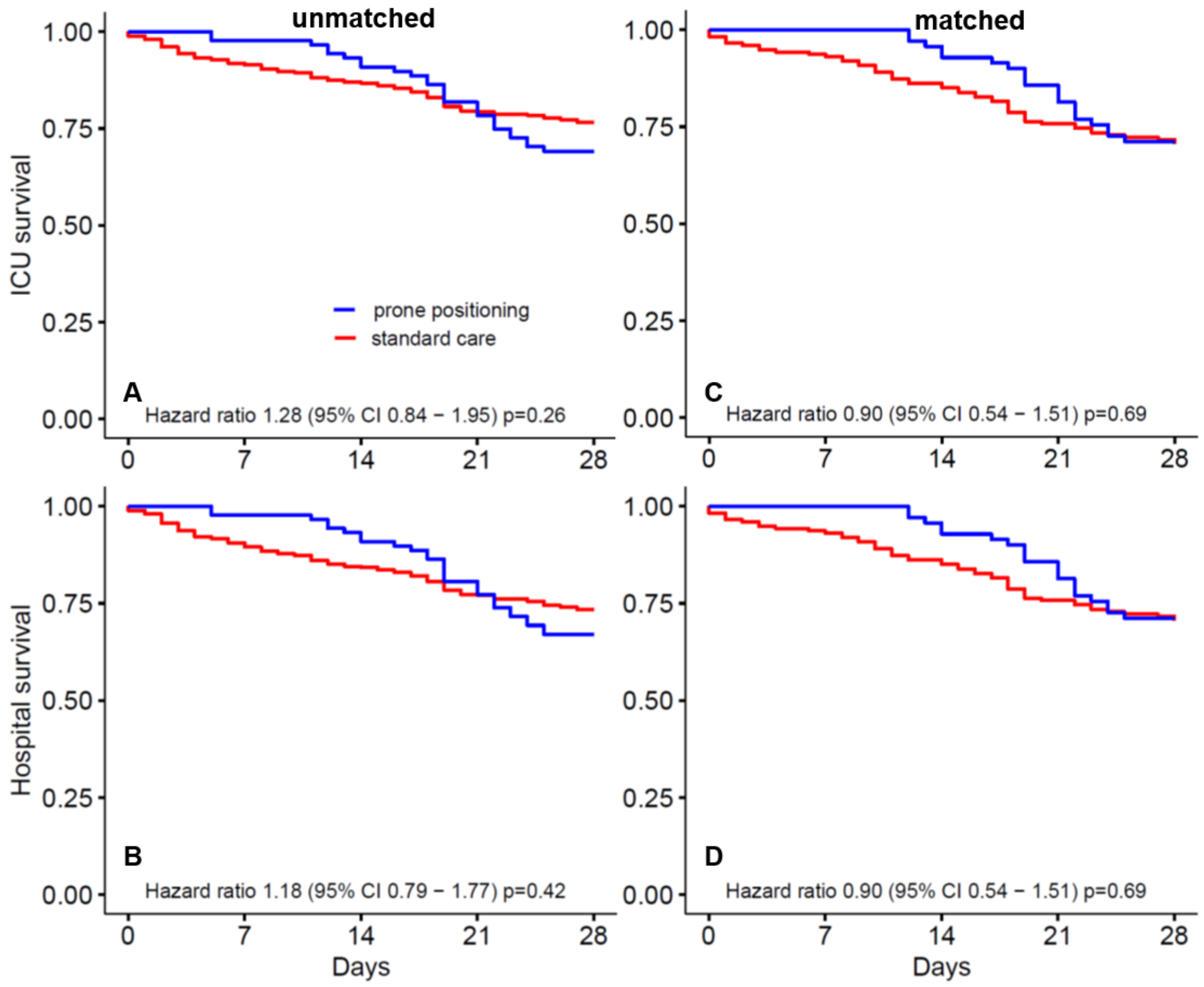

Supplement: Supplementary file 1 [file jcm-11-06988-s001.zip › jcm-2000158-supplementary.pdf]
